# Supplementary material for: Bioenergetics of pollen tube growth in Arabidopsis thaliana revealed by ratiometric genetically encoded biosensors
Source: Nat Commun. 2022 Dec 19;13:7822. doi: 10.1038/s41467-022-35486-w (PMC9763403; doi:10.1038/s41467-022-35486-w)
Supplement: Supplementary file 1 — Supplementary Information [file 41467_2022_35486_MOESM1_ESM.pdf]

Supplementary Data

Bioenergetics of pollen tube growth in *Arabidopsis thaliana* revealed by ratiometric in planta genetically encoded biosensors

Liu et al.

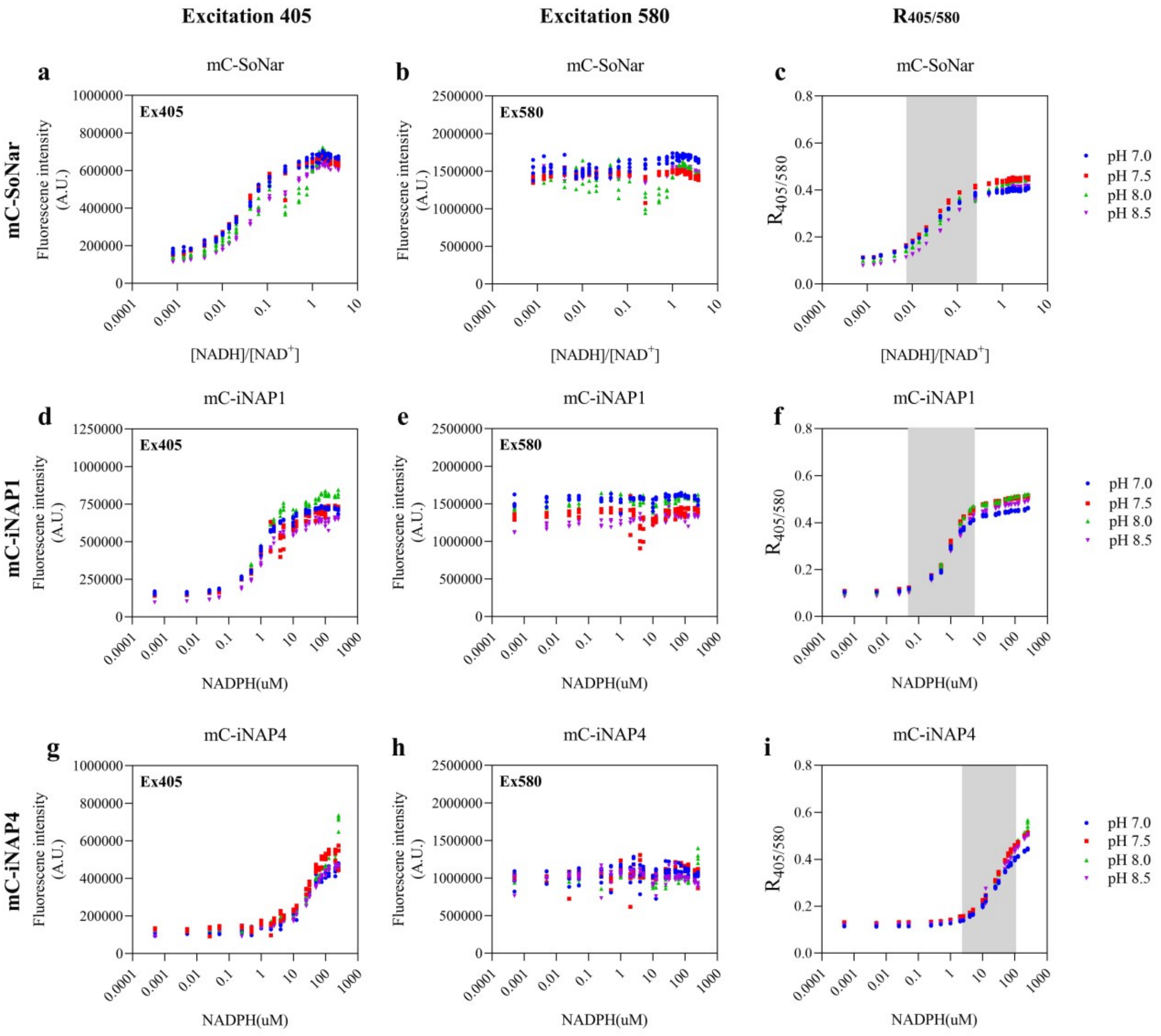

Supplementary Figure 1. **Fluorescence intensities (Ex 405 or Ex 580) and fluorescence ratio ($R_{405/580}$) of purified mCherry-SoNar, mCherry-iNAP1, and mCherry-iNAP4 proteins plotted with various NADPH or NADH/NAD^+ concentrations and pHs.**

The fluorescence intensity readings of **(a-c)** mCherry-SoNar, **(d-f)** mCherry-iNAP1, and **(g-i)** mCherry-iNAP4 at different pHs (7.0, 7.5, 8.0, and 8.5) and various pyridine nucleotide concentrations were measured in a black 96-well microplate using Cytation plate reader. *n* = 4 biologically independent samples; errors bar ± SEM; A.U., arbitrary units.

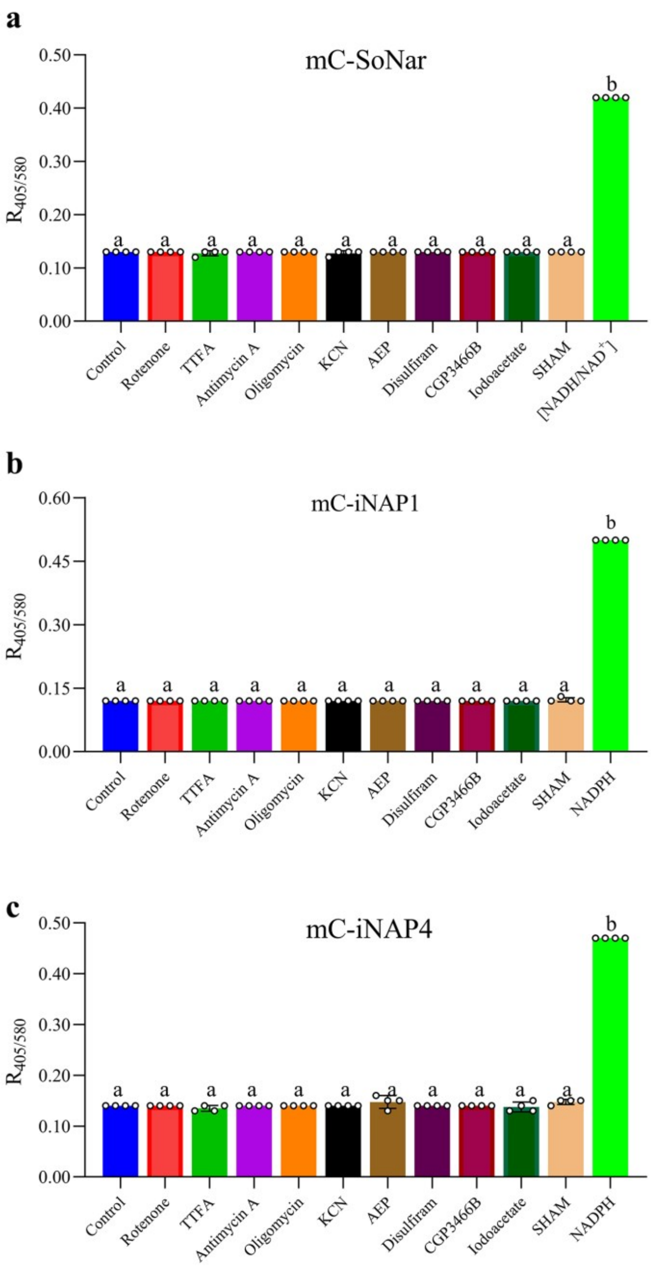

Supplementary Figure 2. **Commonly used inhibitors have no effects on sensor ratios.**
50 µL purified **(a)** mCherry-SoNar, **(b)** mCherry-iNAP1, **(c)** mCherry-iNAP4 proteins at concentration of 0.5 µM were mixed with 50 µL of inhibitors [50 µM rotenone, 100 µM thenoyltrifluoroacetone (TTFA), 10 µM antimycin A, 10 µM oligomycin, 500 µM potassium cyanide (KCN), 90 µM AEP, 30 µM disulfiram, 40 µM CGP3466B maleate, 100 µM iodoacetate, 2 mM salicylhydroxamic acid (SHAM)] in each well of a black 96-well microplate. 100 µM [$NADH/NAD^+$] at 1:1 ratio or 500 µM NADPH were added as positive controls. The fluorescence intensities were collected by the Cytation 1 multi-mode reader. Treatments with significant differences as determined by Tukey's HSD ($P < 0.05$) are indicated with different letters; $n = 4$ biologically independent samples; error bars ± SEM. Exact *p*-values for panel a to c are provided in the source data file.

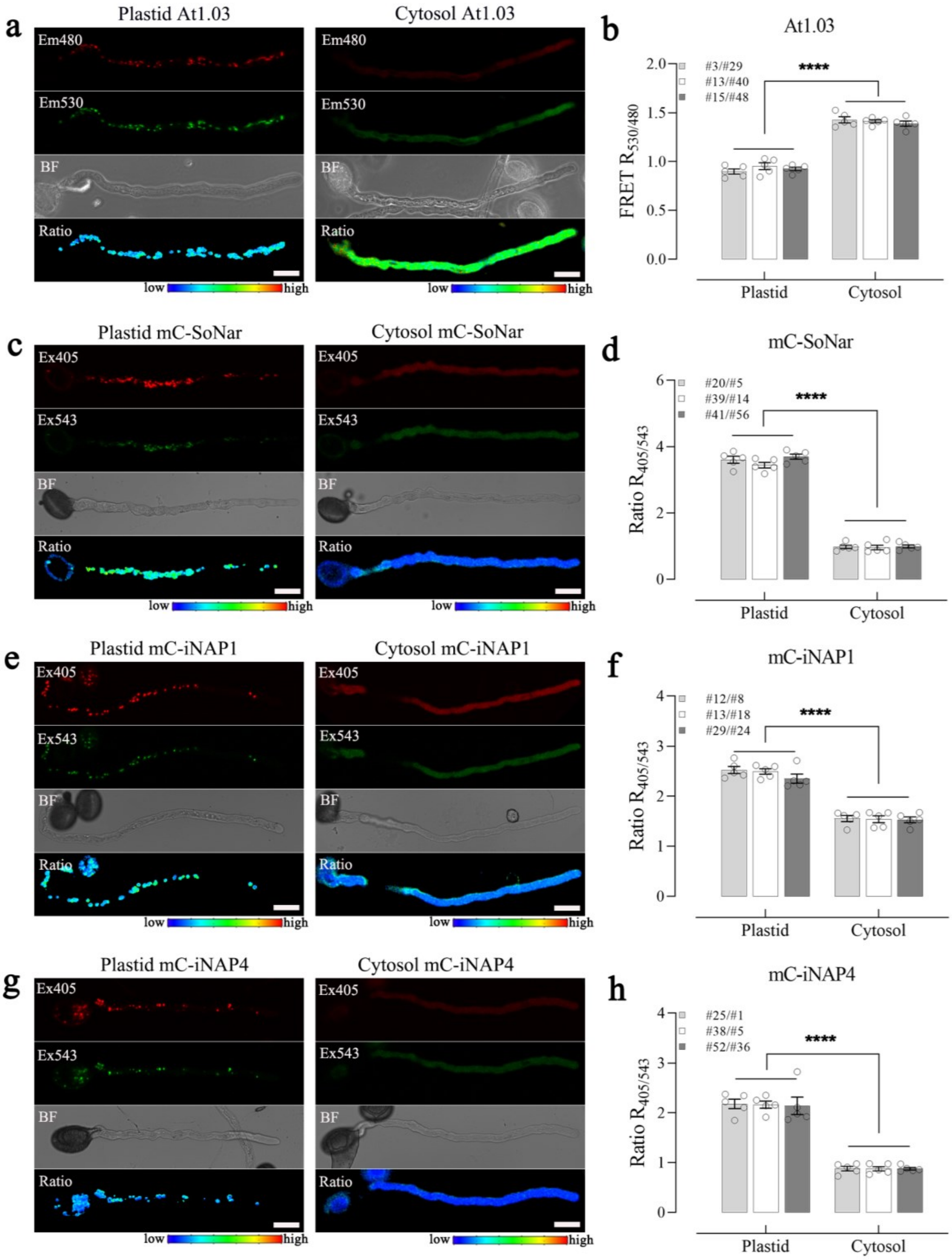

a
Plastid At1.03
Cytosol At1.03
Em480
Em530
BF
Ratio
low
high
b
At1.03
FRET $R_{530/480}$
#3/#29
#13/#40
#15/#48
****
Plastid
Cytosol
c
Plastid mC-SoNar
Cytosol mC-SoNar
Ex405
Ex543
d
mC-SoNar
Ratio $R_{405/543}$
#20/#5
#39/#14
#41/#56
e
Plastid mC-iNAP1
Cytosol mC-iNAP1
f
mC-iNAP1
#12/#8
#13/#18
#29/#24
g
Plastid mC-iNAP4
Cytosol mC-iNAP4
h
mC-iNAP4
#25/#1
#38/#5
#52/#36

Supplementary Figure 3. Overview of pollen tubes expressing various biosensors in three independent lines.

(a, b) The ATP biosensor At1.03 in plastids and the cytosol. *p* value of panel b = 1.130 x 10^{-19}. **(c, d)** The NADH/NAD^+ biosensor mC-SoNar in plastids and the cytosol. *p* value of panel d = 4.358 x 10^{-6}. **(e-h)** The NADPH biosensors, mC-iNAP1 and mC-iNAP4, in plastids and the cytosol. *p* value of panel f = 6.827 x 10^{-16} and *p* value of panel h =2.775 x 10^{-17}. The FRET ratio $R_{530/480}$ was the ratio of fluorescence intensity detected at 526–545 nm (Ex = 458 nm, red channel) over that at 470–507 nm (Ex = 458 nm, green channel). $R_{405/543}$ was obtained from the fluorescence intensity excited at 405 nm (Em = 535 ± 40 nm) in the red channel divided by that excited at 543 nm (Em = 630 ± 60 nm) in the green channel. The ratio images were plotted as pseudocolour HSV with the red representing higher substrate concentration. All biosensors have similar ratios in pollen of three independent lines. Among all lines, #13 (TKTP-At1.03), #40 (Cytosol At1.03), #20 (TKTP-mC-SoNar), #14 (Cytosol mC-SoNar), #29 (TKTP-mC-iNAP1), #24 (Cytosol mC-iNAP1), #52 (TKTP-mC-iNAP4), and #1 (Cytosol mC-iNAP4) were selected for further experiments. Scale bars = 20 µm (*n* = 5 biological independent pollen tubes in each column; error bars ± SEM). Asterisks indicate statistically significant differences (***$P<0.001$, Tukey's HSD test for the same biosensors in three independent lines, and Nested two-sided *t*-test for the comparison of the same biosensor in plastid and the cytosol). mC, mCherry. BF, bright field.

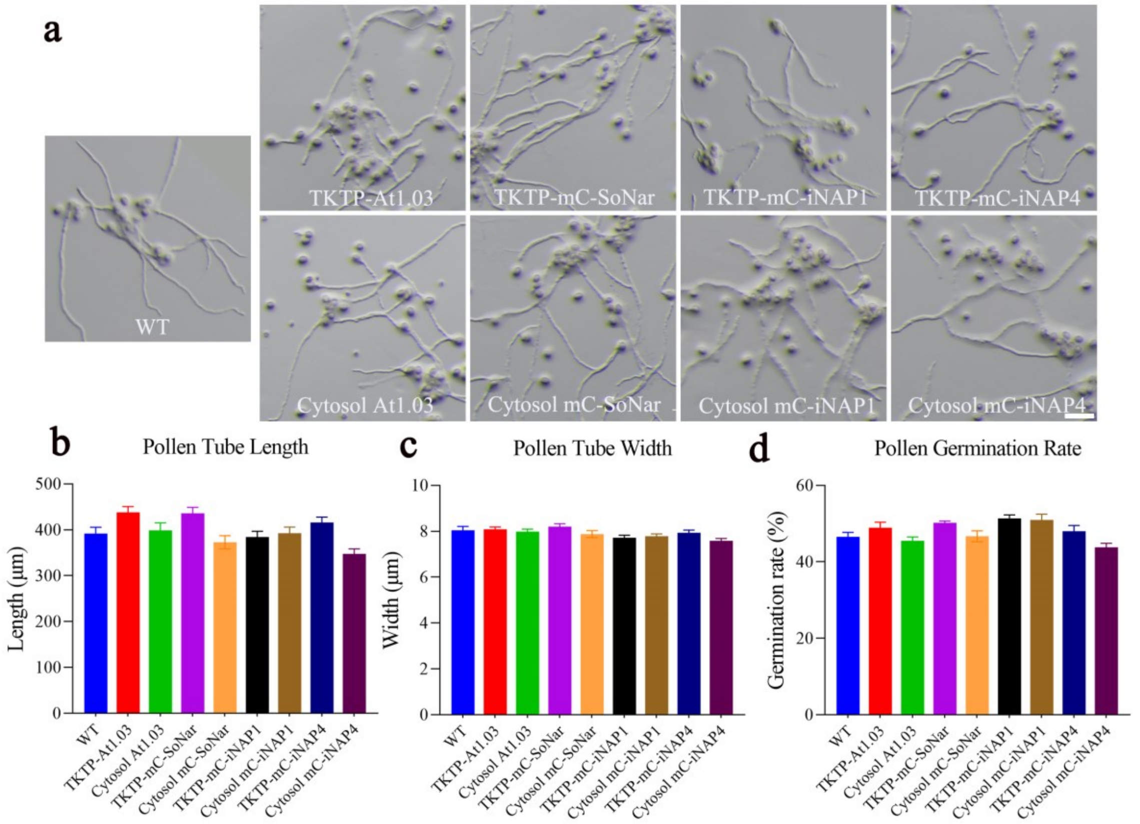

Supplementary Figure 4. Effects of the expression of the mCherry biosensors on pollen tube growth.

Pollen grains of different biosensor lines were germinated on solid pollen germination medium and incubated at 28°C. **(a)** Bright-field images of pollen tubes were taken after 4 h incubation. **(b)** Pollen tube length, **(c)** pollen tube width, and **(d)** pollen germination rates were measured. n = 85 in b and c. For pollen germination rate, at least 500 pollen were counted for each group (error bars ± SEM). Compared to WT pollen, no significant difference was found in the biosensor pollen. p values of panel b = 0.086 (TKTP-At1.03), 0.999 (Cytosol At1.03), 0.111 (TKTP-mC-SoNar), 0.880 (Cytosol mC-SoNar), 0.999 (TKTP-mC-iNAP1), > 0.999 (Cytosol mC-iNAP1), 0.709 (TKTP-mC-iNAP4), and 0.108 (Cytosol mC-iNAP4). p values of panel c = 0.999 (TKTP-At1.03), 0.999 (Cytosol At1.03), 0.915 (TKTP-mC-SoNar), 0.904 (Cytosol mC-SoNar), 0.305 (TKTP mC-iNAP1), 0.571 (Cytosol mC-iNAP1), 0.992 (TKTP-mC-iNAP4), and 0.058 (Cytosol mC-iNAP4). p values of panel d = 0.625 (TKTP-At1.03), 0.992 (Cytosol At1.03), 0.220 (TKTP-mC-SoNar), 0.999 (Cytosol mC-SoNar), 0.063 (TKTP-mC-iNAP1), 0.096 (Cytosol mC-iNAP1), 0.941 (TKTP-mC-iNAP4), and 0.493 (Cytosol mC-iNAP4) (***$P < 0.001$, one-way ANOVA with Dunnett's multiple comparison test). Scale bar = 100 μm. mC, mCherry.

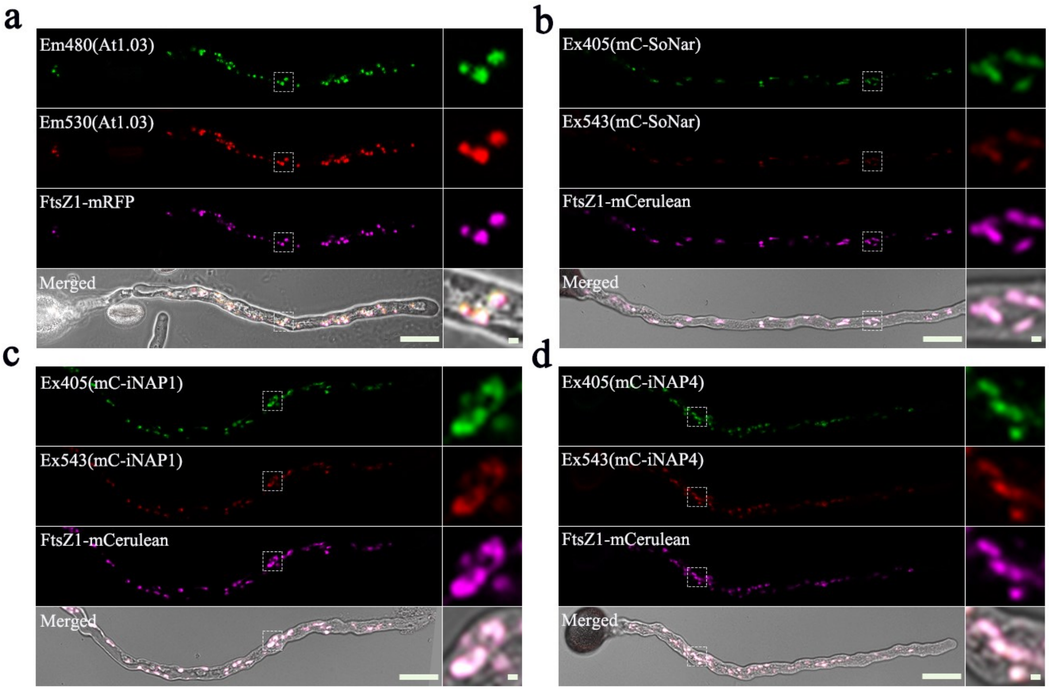

Supplementary Figure 5. Verification of plastid localization of TKTP biosensors.
Plastid localization of (**a)** TKTP-At1.03, (**b)** TKTP-mC-SoNar, (**c)** TKTP-mC-iNAP1, and **(d)** TKTP-mC-iNAP4 was confirmed with plastid marker protein FtsZ1 fused to mRFP or mCerulean fluorophore (violet) in pollen tubes of their corresponding crossing lines. FRET-based At1.03 was excited at 458 nm with double emission at 488 ± 19 nm (green) and 535 ± 10 nm (red), and mRFP was excited at 543 nm with emission of 580 ± 15 nm (purple). mCherry-iNAP/SoNar biosensors were excited at 405 nm (green) and 543 nm (red), and emission was detected at 545 ± 20 nm and 630 ± 60 nm. mCerulean was excited at 458 nm with emission of 478 ± 13 nm (purple). Colocalization experiments for each line were repeated twice and similar results were obtained. Dashed boxes indicate the 5-fold enlarged area. Scale bar = 20 μm (1 μm for the inset). mC, mCherry.

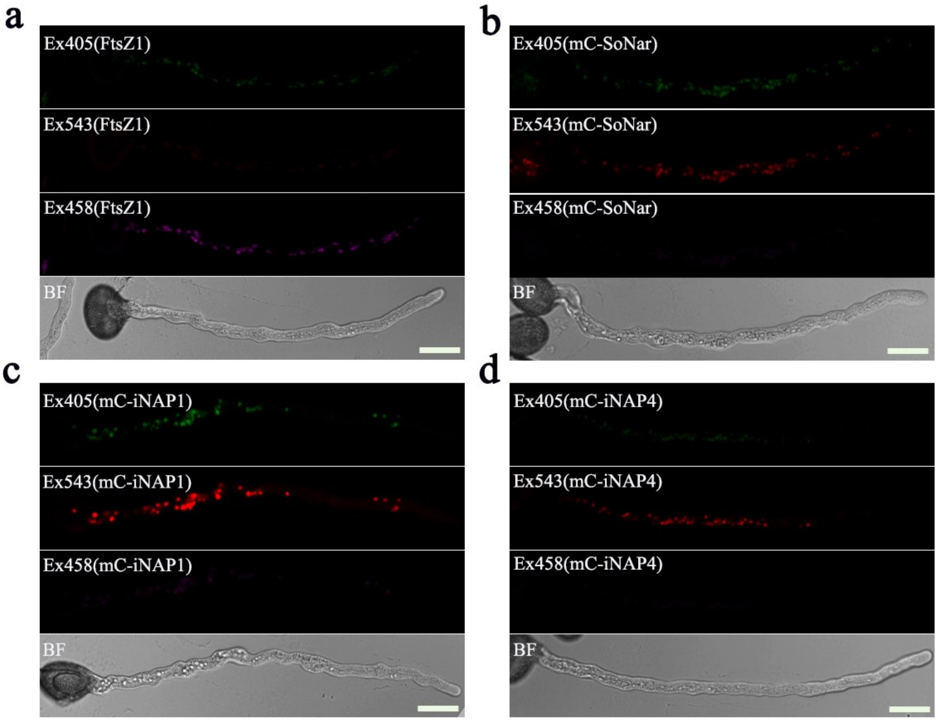

Supplementary Figure 6. Evaluation of possible fluorescence crosstalk between channels.
Pollen tubes of transgenic plants (**a)** FtsZ1-mCerulean, (**b)** TKTP-mC-SoNar, (**c)** TKTP-mC-iNAP1, **(d)** TKTP-mC-iNAP4 were scanned sequentially under the same imaging settings (Ex405 nm/Em545 ± 20 nm (green), Ex543 nm/Em630 ± 60 nm (red), Ex458 nm/478 ± 13 nm (violet)). A little fluorescence bleed-through was excited from mCerulean in the Ex405 detection channel. Fluorescence crosstalk excited by iNAP1, iNAP4, and SoNar in the mCerulean channel was negligible. This experiment was repeated once, and more than 6 pollen were examined for each line. All recorded pollen tubes showed similar results. Scale bar = 20 μm. mC, mCherry. BF, bright field.

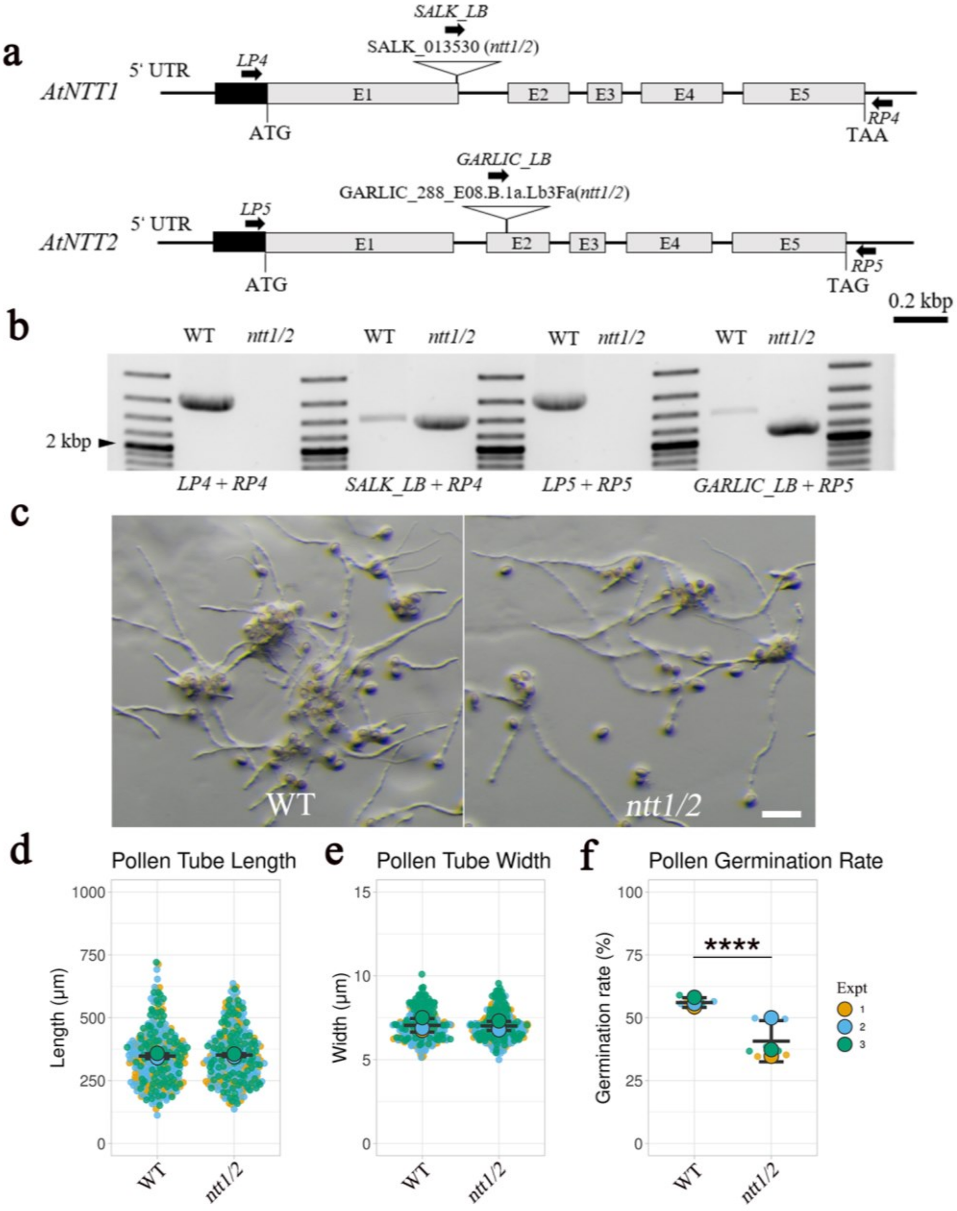

Supplementary Figure 7. Genotyping and in vitro pollen tube growth assay of *ntt1/2 double mutant.*

(a) Schematic representation of T-DNA insertion site of *ntt1/2* double knockout line (SALK_013530, GARLIC_288_E08.b.1a.Lb3Fa) [8]. Numbers in gray boxes represent the exon numbers. UTR, untranslated region. Scale bar = 200 bp. **(b)** Genotyping analysis of *ntt1/2* double knockout line. **(c-f)** In vitro pollen tube growth analysis of WT and *ntt1/2* double knockout line. Pollen tube length, pollen tube width, and pollen germination rate were measured after 4 h pollen incubation. p value of panel d = 0.668, p value of panel e = 0.631, and p value of panel f = 1.259 x 10^{-5}. n = 100 biological independent pollen in each replicate for pollen length and width measurement, and n = 3 for pollen germination rate in each replicate, error bars ± S.D. of mean value of each replicate. Asterisks indicate statistically significant differences (***P＜0.001, two-sided unpaired *t*-test). Scale bar = 100 μm.

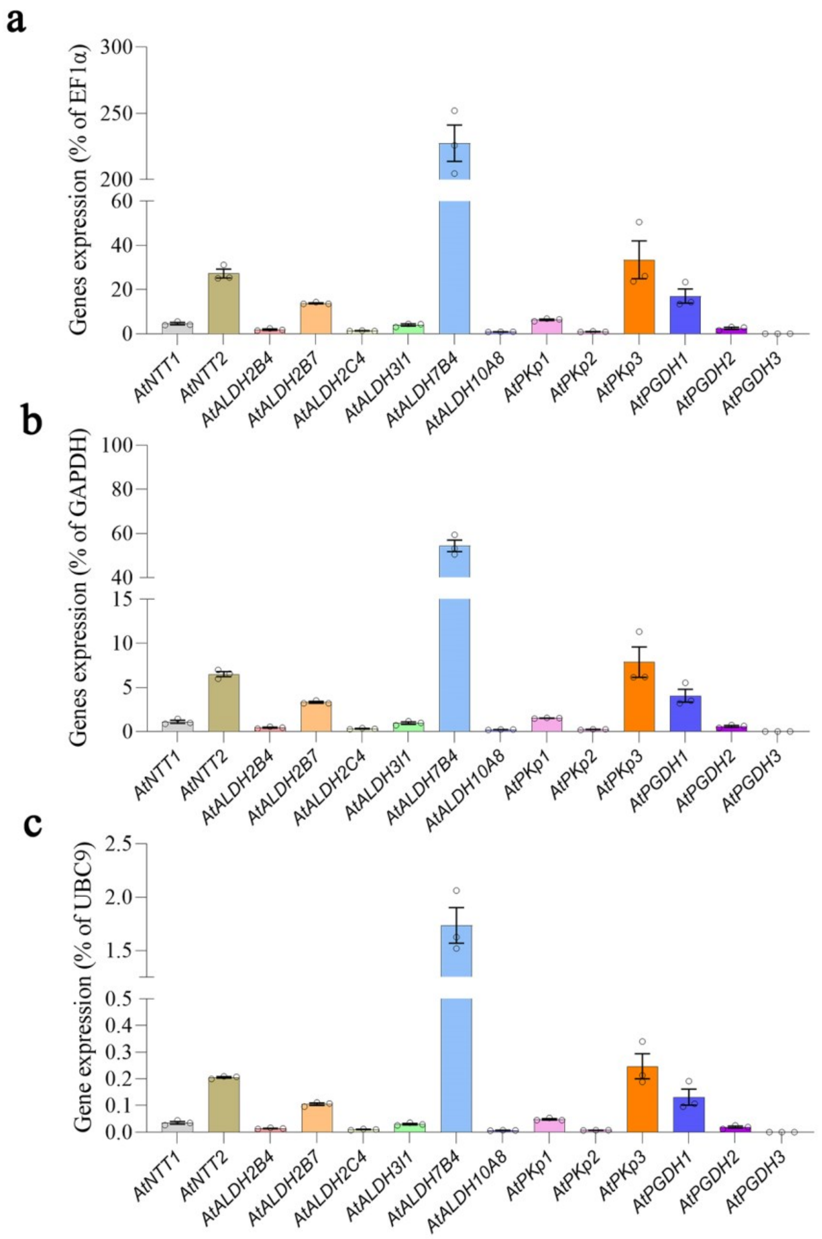

Supplementary Figure 8. RT-qPCR analysis of relative mRNA expression.
(a-c) *EF1α*, *GAPDH*, and *UBC9* were used as internal loading controls for RT-qPCR analysis of *NTT*, *ALDH*, *PKp*, and *PGDH* mRNA expression levels in pollen tubes after 4 h growth. Another batch of biological samples was used here, and the relative expression levels reproduced the data presented in Fig. 3b and Fig. 6j. Error bars ± SEM.

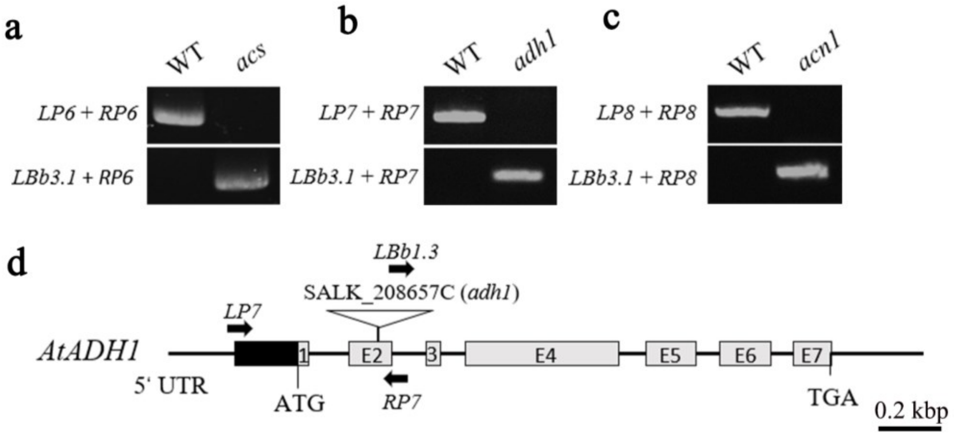

Supplementary Figure 9. Characterization of T-DNA insertion mutants.
Genotyping PCR of the WT, **(a)** *acs*, **(b)** *adh1*, and **(c)** *acn1* T-DNA insertion mutants. **(d)** Schematic representation of T-DNA insertion in *adh1* genes. UTR, untranslated region. The gray number boxes with numbers represent for gene exons. Scale bar = 200 bp.

Supplementary Table 1. Microarray expression levels of *ALDH*, *PGDH*, and *PKp* gene families in Arabidopsis.

Gene Annotation	Subcellular	Locus	Pollen				Other tissues				
			Dry Pollen	0.5h PT	4h PT	SIV PT	8-d seedling	17-d root	17-d rosette	Ovary	Stigma
**ALDH Gene Family (Total 16 members in Arabidopsis genome)**											
ALDH2B4	Mitochondria[80]	AT3G48000	6.41	6.18	6.24	6.31	10.78	9.17	9.61	9.61	9.44
ALDH2B7	Mitochondria[66]	AT1G23800	7.65	7.71	7.69	6.3	5.45	8.34	5.95	4.92	4.71
ALDH2C4	Cytosol[81]	AT3G24503	5.21	5.42	5.02	4.45	9.82	9.1	9.01	7.28	5.74
ALDH3F1	Cytosol[34]	AT4G36250	6.87	6.99	6.54	6.94	8.76	6.83	9.04	9.61	10.1
ALDH3H1	Cytosol[34]	AT1G44170	6.39	6.32	6.03	5.55	7.77	9.27	8	9.22	8.26
ALDH3I1	Chloroplasts[20]	AT4G34240	5.41	5.66	5.89	6.93	7.95	6.27	8.18	7.88	7.83
ALDH5F1	Mitochondria[82]	AT1G79440	5.98	6.36	6.29	6.21	7.4	7.87	8.4	6.6	6.07
ALDH6B2	Mitochondria[80]	AT2G14170	6.76	6.54	6	6.13	10.02	10.06	9.27	9.21	8.85
ALDH7B4	Cytosol[20]	AT1G54100	8.13	8.16	9.13	11.1	10.1	9.88	7.96	9.27	9.23
ALDH10A8	Leucoplasts[21]	AT1G74920	5.84	5.77	6.19	6.02	9.79	10.71	9.84	9.96	9.59
ALDH10A9	Peroxisomes[21]	AT3G48170	7.76	7.74	7.75	7.85	8.71	9.48	8.77	8.88	9.09
ALDH11A3	N.A.	AT2G24270	6.75	6.42	6.61	6.15	10.77	4.88	10.12	10.43	9.89
ALDH12A1	Mitochondria[80]	AT5G62530	6.71	6.6	6.48	6.03	7.65	8.07	8.23	7.47	7.16
ALDH18B1	N.A.	AT2G39800	N.A.	N.A.	N.A.	N.A.	N.A.	N.A.	N.A.	N.A.	N.A.
ALDH18B2	Cytosol[83]	AT3G55610	8.66	8.78	8.8	9.26	8.73	8.45	7.63	7.58	7.25
ALDH22A1	Cytosol[34]	AT3G66658	6.16	6.12	6.15	7.65	6.87	8.2	7.21	7.22	6.74
**PGDH Gene Family (3 members in Arabidopsis genome)**											
PGDH1	Plastid[84]	AT4G34200	7.18	7.22	7	7.55	8.77	10.85	9.7	9.76	8.99
PGDH2	Plastid[84]	AT1G17745	6.63	6.44	6.36	5.89	6.53	10.12	7.57	10.3	9.23
PGDH3	Plastid[84]	AT3G19480	4.7	4.95	4.66	4.14	8.03	3.77	7.6	4.58	4.72
**PKp Gene Family (3 members in Arabidopsis genome)**											
PKp1	Plastid[77]	At3g22960	6.18	5.84	5.92	4.94	9.81	9.82	9.91	8.38	8.57
PKp2	Plastid[77]	At5g52920	4.53	4.56	4.5	4.9	9.31	9.33	9.19	9.4	9.78
PKp3	Plastid[77]	At1g32440	7.45	7.53	7.27	7.88	7.95	7.83	8.16	6.99	7.58

Microarray data were extracted from reference 60. Values shown in the table represent the average of normalized expression levels (log_2) of 3-4 replicates. The average expression levels of all 22809 transcripts on the microarray was ~6.15 (log_2). PT, pollen tube. SIV, semi-in vivo. N.A., Not available.

Supplementary Table 2. Primer sequences

Primer	Sequence
**Primer sequences for mCherry biosensor constructs**	
pRSET-mCherry FW	5′-TAATGAATTCATGTTGAGCAAGGGCGAGGAGGAT-3′
mCherry_(GGSGG)$_2$_G RW	5′-ATTAGGATCCACCTCCTCCAGAACCTCCTCCTCCAGAACCTCCCTTGT ACAGCTCGTCCATGCCG-3′
GG_GGSGG-iNAP/SoNar FW	5′-TAATGGATCCGGAGGAGGAGGTTCTGGAGGAATGAACCGGAAGTGG GGCCT-3′
pRSET-iNAP/SoNar RW	5′-TAATAAGCTTTTAGCCCATCATCTCCTCCCGCC-3′
pENTR- LAT52 FW	5′-GCCGCCCCCTTCACCGGTACCGTCGACATACTCGACTCAGAAGGTAT-3′
pENTR- LAT52 RW	5′-CTTGGGATCGATCGGCATATGTTTAAATTGGAATTTTTTTTTTTGGT-3′
TKTP FW	5′-CATTCATATGATGGCGTCTTCTTCTTCTCT-3’
TKTP RW	5′-ATTACTGCAGCGCAGTCTAGTTTTCTCTAT-3’
mCherry-iNAP/SoNar FW	5′-AGCGCGGATCCGCGACTAGTATGTTGAGCAAGGGCGAGGA-3′
mCherry-iNAP/SoNar RW	5′-CGCCCACCCTTGGGTCTAGATCAGCCCATCATCTCCTCCC-3′
**Primer sequences for FtsZ1 constructs**	
pBI121- LAT52 FW	5′-ACTGTCGACCTCGAGGGTACCATGGCGATAATTCCGTTAGCA-3′
pBI121- LAT52 RW	5′-CTCCATCCCGGGAGCGGTACCGAAGAAAAGTCTACGGGGAGAAGA-3′
FtsZ1 (mRFP) FW	5′-GACCATGATTACGCCAAGCTTGTCGACATACTCGACTCAGAAGGTAT-3′
FtsZ1 (mRFP) RW	5′-CGATCGGGGAAATTCGAGCTCTTTAAATTGGAATTTTTTTTTTTGGT-3′
FtsZ1 (mCerulean) FW	5′-GAGAACACGGGGGACTCTAGAATGGCGATAATTCCGTTAGCA
FtsZ1 (mCerulean) RW	5′-GCCCTTGCTCACCATTCTAGAGAAGAAAAGTCTACGGGGAGAA-3′
FtsZ1-mRFP FW	5′-AAATTCCAATTTAAAGAGCTCATGGCGATAATTCCGTTAGCA-3′
FtsZ1-mRFP RW	5′-GATCGGGGAAATTCGAGCTCTTAGGCGCCGGTGGAGTGGC-3′
FtsZ1-mCerulean FW	5′-AAATTCCAATTTAAAGAGCTCATGGCGATAATTCCGTTAGCA-3′
FtsZ1-mCerulean RW	5′-GATCGGGGAAATTCGAGCTCTTACTTGTACAGCTCGTCCAT-3′
**Primer sequences for the GUS constructs**	
NTT1Pro FW	5′-GACCATGATTACGCCAAGCTTTGGACCTACATATGGGTTCGATT-3′
NTT1Pro RW	5′-ATAAGGGACTGACCACCCGGGCTCTCTATTTCACTCTCTCCCGCA-3′
NTT2Pro FW	5′-GACCATGATTACGCCAAGCTTGGAAGAATCTGAAGTTTTGGAACC-3′
NTT2Pro RW	5′-ATAAGGGACTGACCACCCGGGCTCTCTATCTCTCACGTAGCACACTGA-3′
ALDH3I1Pro FW	5′-GACCATGATTACGCCAAGCTTCATACGATGGCTCATGGCTGTGTAG-3′
ALDH3I1Pro RW	5′-ATAAGGGACTGACCACCCGGGGACTTGGGTTCAGTTAAAAATCTC-3′
ALDH10A8Pro FW	5′-GACCATGATTACGCCAAGCTTGGGCGTTGAATCGGACACACA-3′
ALDH10A8Pro RW	5′-ATAAGGGACTGACCACCCGGGTGTTCTTATCTGAATCTAGAGCTCCG-3′
ALDH2B7Pro FW	5′-GACCATGATTACGCCAAGCTTAGCTAGTCTTCCACCAGTGC-3′
ALDH2B7Pro RW	5′-ATAAGGGACTGACCACCCGGGCTTGATGCCATGGCTAACTTT-3′
ALDH7B4Pro FW	5′-GACCATGATTACGCCAAGCTTTCCCACTACTGAATTGACCTTCA-3′
ALDH7B4Pro RW	5′-ATAAGGGACTGACCACCCGGGCTCTGCGCAAAAATTCACCCCA-3′
**Primer sequences for genotyping T-DNA insertion lines**	
SALK_083518C_LP1(ntt1-1)	5′-TCTCAGCAGAACTTCCTTTCG-3′
SALK_083518C_RP1(ntt1-1)	5′-TTGACATTGGCCACATTACAC-3′
SALK_023159C_LP2(ntt1-2)	5′-CACAAAGAGAGAAGCACCGAG-3′
SALK_023159C_RP2(ntt1-2)	5′-CTTTCCATCGTTCCCATCTTC-3′
SALK_031126C_LP3(ntt2)	5′-TCCCTCGTTTTCATCAACAAC-3′
SALK_031126C_RP3(ntt2)	5′-CTTCTCTCCTTGTGGGAAACC-3′
ntt1/2_LP4	5′-TTTCTTCTGTGTATCTGCGGGAGAGAGTG-3′ [8]
ntt1/2 RP4	5′-CTTTCTTTCCCCCCCAACAAAACCAAATA-3′ [8]
SALK_LB	5′-ACTCAACCCTATCTCGGGCTATTC-3′ [8]
ntt1/2 LP5	5′-TCTCTTCTCCTCTCTACCCAGAGC-3′ [8]
ntt1/2 RP5	5′-CCAAATCCCAAAACCCTTTTATTCATC-3′ [8]
GARLIC_LB	5′-TAGCATCTGAATTTCATAACCAATCCGATACAC-3′ [8]
SALK_015522C_LP6(acs)	5′-GGCAAGTGCAATAAGCTGATC-3′
SALK_015522C_RP6(acs)	5′-TGCCGGATATTATTTCAGTGG-3′

SALK_208657C_LP7 (adh1)	5′-AAACCAAATTATGCATTACGAGC-3′
SALK_208657C_RP7 (adh1)	5′-TCCCAGAAGTAAACATCGGTG-3′
SALK_009373C_LP8(acn1)	5′-TCGAGGTGCGTATATAATGGC-3′
SALK_009373C_RP8(acn1)	5′-TTTTCCAGTTGCTGTCTTTGG-3′
SALK_LBb1.3	5′-ATTTTGCCGATTTCGGAAC-3′
**Primer sequences for qRT-PCR analysis**	
EF1α_qPCR FW	5′-ACCACATGATTGAGAGGTCC-3′
EF1α_qPCR RW	5′-GCATCTCAACAGACTTGACC-3′
GAPDH qPCR FW	5′-CTCTTCGGTGAGAAGCCAGT-3′
GAPDH qPCR RW	5′-CAGCAGCCTTGTCTTTGTCA-3′
UBC9 qPCR FW	5′-CATCGGATAGCCCTTATTCTG-3′
UBC9 qPCR RW	5′-TGGAACACCTTCGTCCTAAAA-3′
NTT1_qPCR FW	5′-GCTTCTCAGCCAATACGTATTC-3′
NTT1_qPCR RW	5′-GCCGCCAAACAATATTAGAGAG-3′
NTT2_qPCR FW	5′-GTTCATGATCCTTACATTCGGC-3′
NTT2_qPCR RW	5′-TGAGAGTGTTAAACTGTCCCTC-3′
ALDH2B4_qPCR FW	5′-TGGACAGATCATACCGTGGA-3′
ALDH2B4_qPCR RW	5′-GCATAGAAAGCCGTGAGAGG-3′
ALDH2B7_qPCR FW	5′-CATGCTCATAGCAACAGACGA-3′
ALDH2B7_qPCR RW	5′-CAGCAGCTAAACCGTACCTTG-3′
ALDH2C4_qPCR FW	5′-GATCAACACGGTTTCGAGGT-3′
ALDH2C4_qPCR RW	5′-GCATAACGACGGATTTGGTT-3′
ALDH3I1_qPCR FW	5′-GACGTATCAGCAGGAGGAATTA-3′
ALDH3I1_qPCR RW	5′-GAGAATTTCCCATGGTAAGCAC-3′
ALDH7B4_qPCR FW	5′-TGGAAACAGTACATGCGTCGAT-3′
ALDH7B4_qPCR RW	5′-CCTTGCGCTAGAGGTAACTCGTT-3′
ALDH10A8_qPCR FW	5′-GAATAAAGGGAAAGATTGGGCC-3′
ALDH10A8_qPCR RW	5′-AGCAACATCATCCATATCCCAT-3′
PKp1 qPCR FW	5′-TCACTGGTCTCCCGATGT-3′
PKp1 qPCR FW	5′-ACCTCTTGATTTCAGTAACGA-3′
PKp2 qPCR FW	5′-AGTCACTATCGTCCTTCCG-3′
PKp2 qPCR RW	5′-CTGTACGATTGCTATTTCCTC-3′
PKp3 qPCR FW	5′-CTACCTGTCAGAACCTCGG-3′
PKp3 qPCR RW	5′-CCTTTGCATTATTCTTCTCTG-3′
PGDH1 qPCR FW	5′-GTTGGGAGAATTGCACCGAG-3′
PGDH1 qPCR RW	5′-AGAATCAACAACACACCAAACTAGAG-3′
PGDH2 qPCR FW	5′-GGACAGGTTGGTAACATACTTGG-3′
PGDH2 qPCR RW	5′-CATCAACTCCAATCGCCATTATC-3′
PGDH3 qPCR FW	5′-CTCTGTTTTTCCCATCCGTCAG-3′
PGDH3 qPCR RW	5′-GCCTAGCTTCTCCGTTACGAG-3′

Supplementary Table 3. Promoter regions for histochemical GUS staining assay

Promoter region	References
NTT1Pro (1.5 kb)	[8]
NTT2Pro (1.5 kb)	[8]
ALDH3I1 (0.9 kb)	[85]
ALDH10A8 (1.3 kb)	[24]
ALDH7B4 (0.8 kb)	[21]
